# Minimally invasive osteosynthesis using a dynamic compression angle‐stable interlocking nail in feline transverse femoral fractures: an ex vivo study

**DOI:** 10.1111/jsap.70059

**Published:** 2025-11-21

**Authors:** M. Nobile, R. Barroco Neto, A. L. C. Carrera, T. A. S. S. Rocha, V. S. V. dos Dias, B. W. Minto, L. G. G. G. Dias

**Affiliations:** ^1^ Universidade Estadual Paulista Julio de Mesquita Filho São Paulo Brazil; ^2^ Universidade Federal de Jatai Jataí Brazil; ^3^ Animals Veterinary Clinic São Paulo Brazil

## Abstract

**Objective:**

To evaluate the effectiveness of interfragmentary compression in feline femoral transverse fractures treated with a dynamic compression angle‐stable interlocking nail implanted via minimally invasive nail osteosynthesis.

**Methods:**

Thirty femurs from 15 feline cadavers were allocated into three groups according to fracture location (*n* = 10 per group): proximal (GPD), mid‐ (GMD) and distal diaphysis (GDD). Radiographic and computed tomography scans were performed preoperatively for surgical planning and measurement of the anatomical lateral distal femoral angle, anatomical lateral proximal femoral angle and femoral torsion angle. Standardised transverse osteotomies were created per group allocation and stabilised using dynamic compression angle‐stable interlocking nail implanted via minimally invasive nail osteosynthesis technique. Postoperative imaging assessed residual fracture gap and femoral angles. Data were analysed using one‐way ANOVA with Tukey’s post hoc test, and effect sizes were estimated using generalised eta squared (ges).

**Results:**

All groups achieved effective interfragmentary compression, with residual fracture gaps <1 mm (range: 0 to 0.3 mm). The anatomical lateral distal femoral angle remained consistent before and after osteosynthesis. The anatomical lateral proximal femoral angle remained unchanged in proximal diaphysis and distal diaphysis but showed a moderate increase in mid‐diaphysis (ges = .445). The femoral torsion angle increased in all groups postoperatively, with large, moderate and small effect sizes in proximal diaphysis (ges = .655), mid‐diaphysis (ges = .385) and distal diaphysis (ges = .213), respectively.

**Clinical significance:**

Dynamic compression angle‐stable interlocking nail provided consistent interfragmentary compression. However, deviations in the transverse plane were observed following the minimally invasive nail osteosynthesis approach. These findings support dynamic compression angle‐stable interlocking nail as a promising option for stabilising femoral transverse fractures in cats.

## INTRODUCTION

The rationale behind minimally invasive osteosynthesis (MIO) has gained prominence in recent years, particularly in clinical practice, due to growing recognition of the role of periosteal soft tissues and vascular structures adjacent to the fracture site in promoting bone healing and enhancing osteogenesis (Guiot & Déjardin, 2011; Johnston et al., 2017; Maritato & Barnhart, 2020). One specific MIO technique is the use of interlocking nails through minimally invasive nail osteosynthesis (MINO), which aims to provide a bridging construct and relative stability for comminuted fractures, yielding satisfactory clinical and biomechanical outcomes (Déjardin et al., 2020; Mund et al., 2023). However, in cases of long bone transverse fractures, anatomical reconstruction and interfragmentary compression are preferred to facilitate primary bone healing for both human and veterinary patients. In such scenarios, dynamic compression plates (DCP) or limited contact DCP (LC‐DCP), in association or not with an intramedullary rod, remain the gold‐standard method of fixation due to their ability to provide rigid stability and promote direct bone healing (Matres‐Lorenzo et al., 2016; Tseng et al., 2025; Ya’ish et al., 2011).

Thus, the surgical approach for achieving interfragmentary compression has traditionally relied on open reduction and internal fixation (ORIF) techniques, which, despite providing superior mechanical resistance and effective load sharing between bone and implants, can result in damage to soft tissues and periosteal vascular structures (Garofolo & Pozzi, 2013; Johnston et al., 2017). In recent years, however, research has increasingly focused on integrating the principles of mechanical stability with biological surgical strategies. Accordingly, MIO techniques employing implants that enable dynamic compression have been used (Jirangkul et al., 2024; Xue et al., 2016). High‐strain fractures are generally thought to require rigid fixation; however, some veterinary patients may still achieve bone healing under relative stability; consequently, callus formation is expected during the process (Tanveer et al., 2025). From a biological perspective, the MINO technique offers advantages in terms of fracture reduction and bone realignment, while preserving the vascular integrity of the surrounding tissues (Déjardin et al., 2020). Nevertheless, until recently, no interlocking nail system combining dynamic compression with angle‐stable locking had been available for veterinary use. The recent development of a dynamic compression angle‐stable interlocking nail (DCASIN) represents a promising solution (Dias et al., 2025).

Considering the potential to simultaneously apply dynamic compression and a minimally invasive approach for the treatment of transverse fractures, the DCASIN may represent a promising interlocking implant option (Dias et al., 2025). This model has demonstrated favourable outcomes in laboratory‐based mechanical testing (Dias et al., 2025); however, no previous studies have investigated its implantation using the MINO technique or its effectiveness in achieving interfragmentary compression in feline femoral transverse fractures. Therefore, the objective of this study was to evaluate the feasibility of DCASIN implantation via the MINO technique in ex vivo feline femoral transverse fractures, with particular emphasis on the surgical approach, angular deviations and interfragmentary compression capacity, assessed by measuring the residual fracture gap. The authors hypothesised that the DCASIN can be effectively implanted via the MINO approach and provide satisfactory bone‐to‐bone contact.

## MATERIALS AND METHODS

This study aimed to evaluate the implantation technique of the DCASIN using the MINO approach, as well as its ability to provide interfragmentary compression in ex vivo models of feline femoral transverse fractures. The research protocol was approved by the Institutional Animal Care and Use Committee of São Paulo State University (protocol number 1567/21). Feline cadavers were used in the study, with causes of death unrelated to the objectives of this research and with informed consent obtained from the owners.

### Study design and inclusion criteria

A total of 15 feline cadavers (*Felis catus*) were used in this prospective study. All animals had died from causes unrelated to the objectives of the research and were obtained from the Veterinary Teaching Hospital of BLINDED. Inclusion criteria consisted of a body weight between 3.5 and 5.0 kg and a good general post‐mortem condition at the time of evaluation. Exclusion criteria included a history of femoral fractures, soft tissue injuries in the hindlimbs and macroscopic evidence of tumours, neoplasia or disfiguring injuries in the pelvic limbs. Immediately after death, all cadavers underwent radiographic examination in craniocaudal and mediolateral projections prior to inclusion to confirm the integrity of both femurs and to plan the experimental osteotomy sites simulating transverse fractures. Sequentially, they were frozen at −20°C and preserved with 0.9% NaCl solution to maintain anatomical characteristics and tissue integrity until the time of the surgical procedure.

### Outcomes

After inclusion, the 15 feline cadavers were randomly assigned to three groups according to the location of the experimentally induced diaphyseal transverse femoral fracture: proximal diaphysis (GPD), mid‐diaphysis (GMD) and distal diaphysis (GDD). Each group comprised five cadavers (10 femurs), totalling 30 femurs for the entire experiment.

**FIG. 1:**
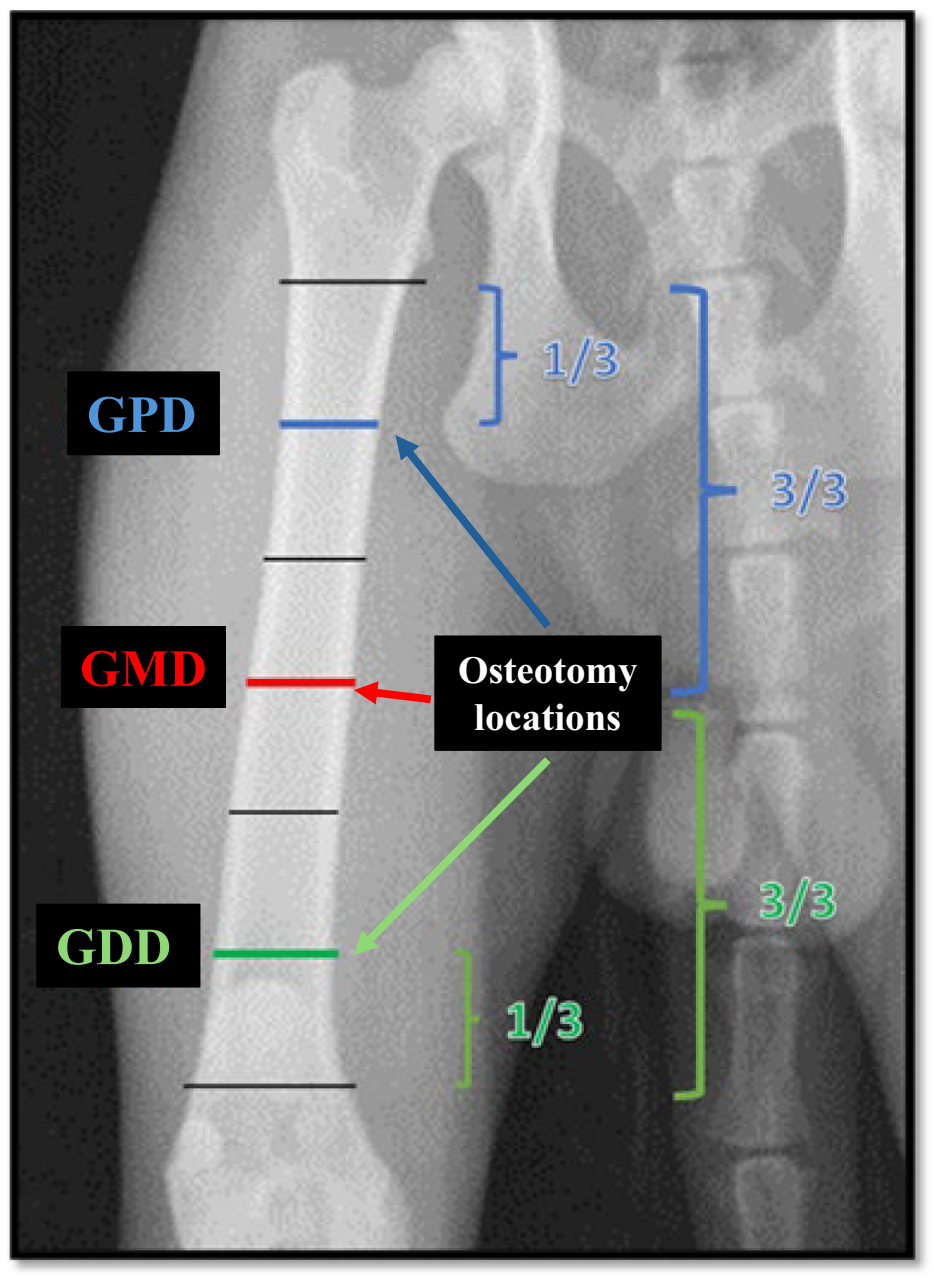
Schematic representation of the planning and determination of femoral osteotomy sites in feline cadavers, simulating transverse fractures in three experimental groups: proximal diaphysis (GPD), mid‐diaphysis (GMD) and distal diaphysis (GDD).

Transverse fractures were created using an oscillating linear saw (Stryker Sabo 2 electric system; Stryker Corporation, Kalamazoo, MI, USA) with a blade of 0.38 mm thickness and 0.63 mm kerf, through a medial surgical approach to the femur, thereby preserving the lateral surface for subsequent MINO implantation. Osteotomy sites were defined based on the radiographic measurements previously obtained (Fig. 1). The diaphysis was delimited and measured to determine the precise location of each osteotomy. In the GMD group, osteotomy was performed at the midpoint of the diaphyseal length. For the GPD group, the proximal half of the diaphysis was divided into three equal parts, and the osteotomy was performed at the junction between the first and second thirds. Similarly, in the GDD group, the distal half of the diaphysis was divided into three equal segments, and the osteotomy was performed at the junction between the second and third thirds.

Osteotomies were performed based on individualised measurements obtained during the planning phase for each animal, using anatomical landmarks such as the greater trochanter proximally and the origin of the trochlear groove distally to determine the osteotomy site in millimetres. Following osteotomy execution, the femurs were radiographically evaluated in craniocaudal and mediolateral projections to subjectively verify the consistency of osteotomy locations in accordance with group allocation.

### Radiographic and tomographic evaluations and measurements

All cadavers underwent radiographic evaluation at three time points: pre‐osteotomy, post‐osteotomy and postoperative following MINO with DCASIN implantation. The pre‐osteotomy evaluation was used for sample inclusion, osteotomy site measurement, surgical planning and baseline bone angle assessment. The post‐osteotomy evaluation served to verify the consistency of osteotomy location according to group allocation. Postoperative images were used to measure bone angles after implant placement. For all time points, craniocaudal and mediolateral radiographic projections were acquired using standardised anatomical landmarks for proper positioning, as described by Petazzoni and Jaeger (2008). The femurs were kept parallel to the examination table and radiographic plate, with a 1‐in. magnification marker to ensure accurate measurements.

Subsequently, the cadavers were submitted to computed tomography (CT) scanning using a 16‐slice GE Revolution ACT scanner (GE Healthcare, 3000 North Grandview Boulevard, Waukesha, WI 53188, USA), with the animals positioned in dorsal recumbency and hindlimbs extended caudally. CT scans were performed at the pre‐osteotomy and postoperative stages. The femurs were reconstructed in three dimensions using RadiAnt DICOM Viewer software (version 2025.2, Medixant, ul. Promienista 25, 60‐288 Poznań, Poland) and positioned according to the methodology described by Petazzoni and Jaeger (2008) for angular measurements.

**FIG. 2:**
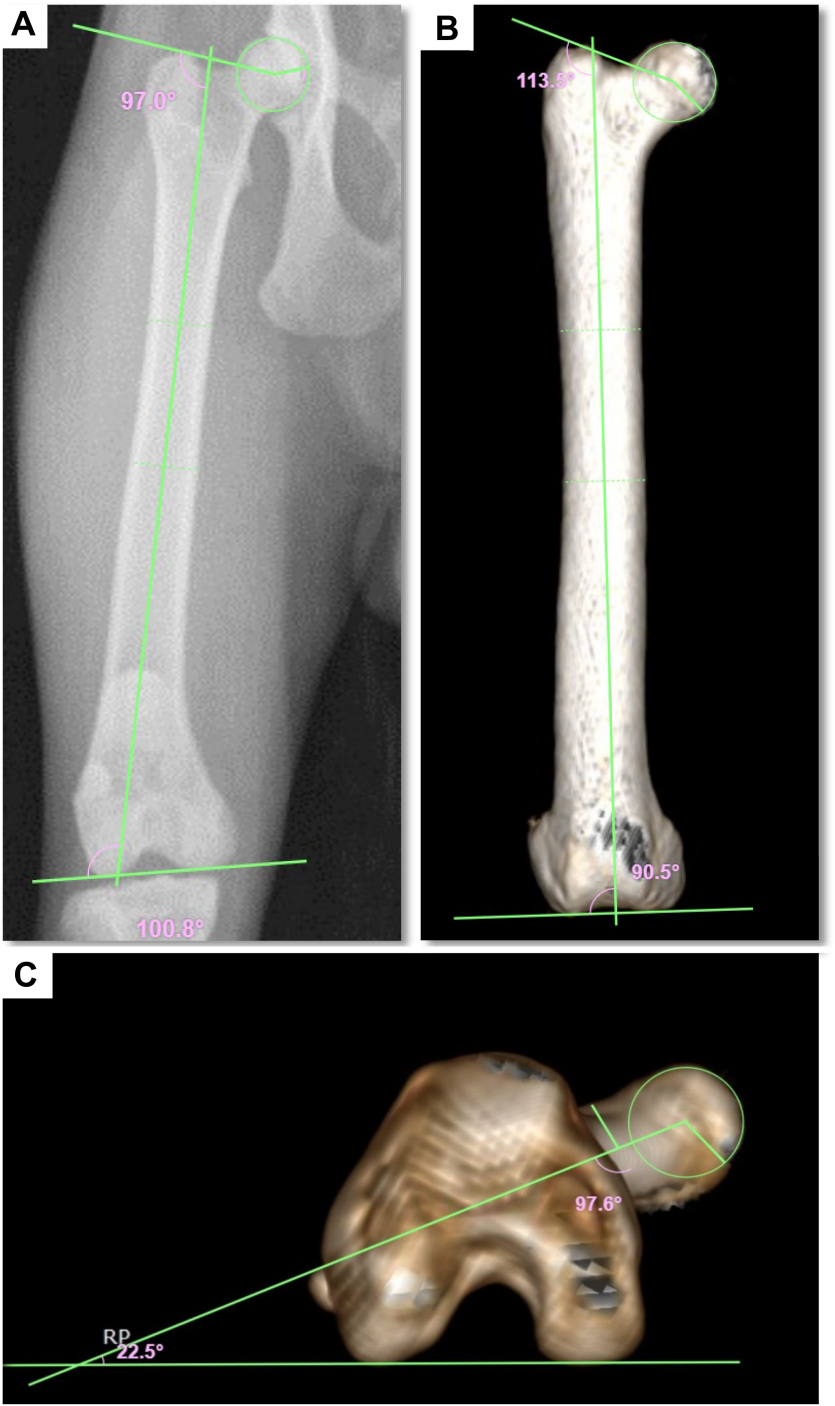
Radiographic (A) and three‐dimensionally reconstructed computed tomographic images (B and C) used for measurement of feline femoral angles (ex vivo), pre‐osteotomy. (A, B) Frontal plane measurements in craniocaudal projections, showing the anatomical lateral proximal femoral angle (aLPFA) and anatomical lateral distal femoral angle (aLDFA). (C) Transverse plane measurement in a distal‐to‐proximal view, showing the femoral torsion angle (FTA).

Radiographic and CT images obtained at the pre‐osteotomy and postoperative time points were used for objective measurement of bone alignment to assess the influence of MINO with DCASIN on deviations in the frontal and transverse planes. The anatomical lateral proximal femoral angle (aLPFA), anatomical lateral distal femoral angle (aLDFA) and femoral torsion angle (FTA) were measured according to standardised protocols (Petazzoni & Jaeger, 2008) (Fig. 2). Each angle was compared between the pre‐osteotomy and postoperative values.

**FIG. 3:**
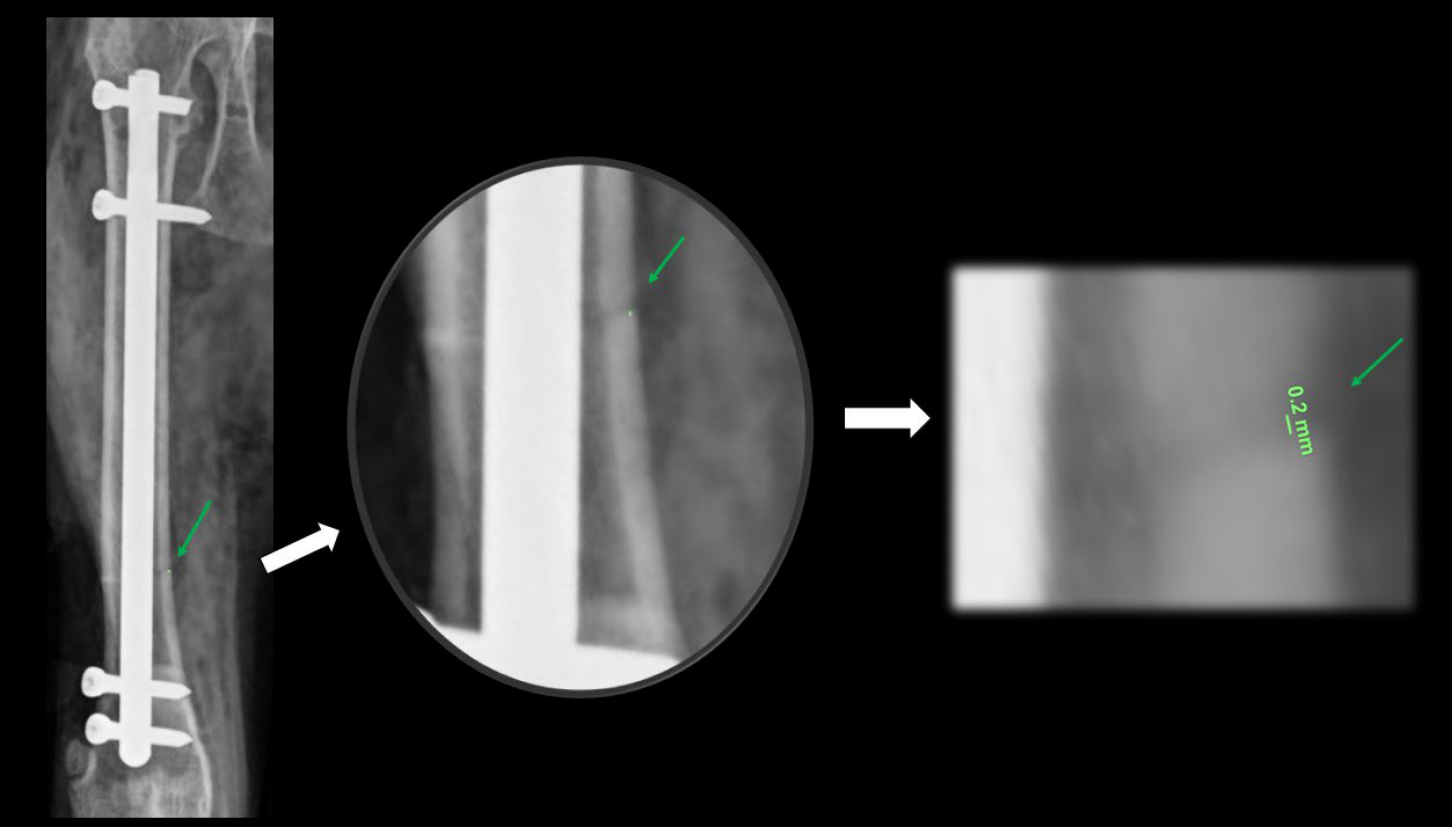
Measurement of the residual fracture gap in a craniocaudal projection following minimally invasive osteosynthesis using a dynamic compression angle‐stable interlocking nail in a feline femoral transverse fracture. The gap was measured as a straight line between the proximal and distal cortices at the widest point between the femoral fragments.

Postoperative images were also used to measure the residual fracture gap in order to assess the achievement of interfragmentary compression. The fracture gap was measured in both craniocaudal and mediolateral projections as a straight line between the proximal and distal cortices at the widest point between the femoral fragments (Fig. 3). For each animal, the largest gap value obtained from either projection was considered for analysis. Ideally, the fracture should be entirely compressed, and a fracture gap of 1 mm is sufficient to affect construct stiffness (Oh et al., 2010). Therefore, the residual gap was classified as ideal when equal to 0 mm, satisfactory when >0 mm and <1 mm and unsatisfactory when ≥1 mm. All measurements were performed using VPop Pro software (VETSOS Education Ltd., Shrewsbury, United Kingdom) by a single evaluator with expertise in veterinary orthopaedic surgery.

### Treatment – MINO Using DCASIN

**FIG. 4:**
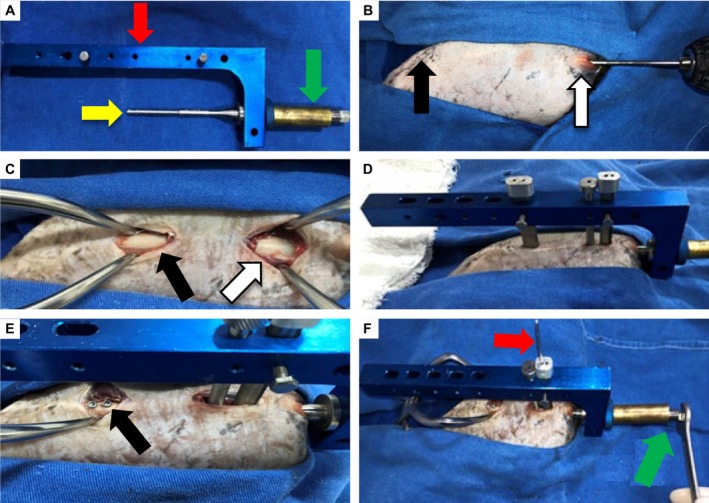
Surgical technique for minimally invasive nail osteosynthesis (MINO) using the dynamic compression angle‐stable interlocking nail (DCASIN). (A) The implant assembly consisted of the DCASIN (yellow arrow), external implantation guide (EIG) and compression device (CD). (B) The medullary canal was milled using drills of increasing diameters in a proximodistal direction, with the greater trochanter as the proximal reference (white arrow) and the patella as the distal landmark (black arrow). (C) Following the MINO approach, the proximal (white arrow) and distal (black arrow) metaphyseal regions were exposed. (D) Insertion of the DCASIN into the medullary canal using the EIG and CD, highlighting the perforation sites. (E) Both distal angle‐stable screws were locked into the femur (black arrow). (F) Execution of the compression technique, showing placement of the 2.0‐mm Steinmann pin (red arrow) in the proximal aspect of the oblong hole, followed by threading of the primary screw into the CD (green arrow) to achieve interfragmentary compression.

The DCASIN model, as well as the dynamic compression technique and its execution, was consistent with the methodology previously described by Dias et al. (2025). The nail was manufactured from 316 L stainless steel by ProtomedVet (São Paulo, São Paulo, Brazil) and featured two angle‐stable threaded locking holes in the proximal and distal regions. Additionally, an oblong hole measuring 7 mm in longitudinal length was positioned proximally between the two locking holes, allowing for Steinmann pin insertion and implementation of the compression technique. In conjunction with the DCASIN, the use of an external implantation guide (EIG) and a compression device (CD), which allowed linear displacement of 7 mm, was required, in accordance with the model’s original specifications (Dias et al., 2025) (Fig. 4A). For this study, the nails used had diameters of 4.0, 4.5 or 5.0 mm, and lengths of 70, 90 or 110 mm, selected based on preoperative planning for each femur. Nail diameter was standardised to approximately 75% of the femoral medullary canal isthmus, as determined from preoperative radiographs, while length was maximised to ensure that both nail extremities reached the femoral metaphyseal regions (Déjardin et al., 2020). The screws used had a total diameter of 2.0 mm, a core diameter of 1.5 mm and sufficient length to achieve bicortical positioning. The Steinmann pin had a diameter of 2.0 mm, and a 1.5 mm diameter drill bit was used for all perforations.

Osteosynthesis was performed by a single surgeon with expertise in veterinary orthopaedics. The femoral medullary canal was reamed using sequentially larger drill bits – 2.0, 3.0, 4.0 and 5.0 mm in diameter – depending on the size of the selected nail (Fig. 4B). Reaming was discontinued once drilling with the same diameter as the nail had been completed for both the proximal and distal bone fragments. The MINO approach was performed according to the technique previously described by Lucena et al. (2023). The proximal and distal metaphyseal regions were exposed for screw fixation, without opening or manipulating the fracture site (Fig. 4C). The DCASIN was then inserted into the medullary canal using the EIG and CD (Fig. 4D), achieving manual external fracture reduction and allowing for screw insertion from the lateral aspect of the femur.

Once the nail was fully inserted, the distal screws were locked bicortically using threaded angle‐stable fixation (Fig. 4E). A Steinmann pin was positioned in the proximal aspect of the oblong hole located in the nail’s proximal region. Next, the primary screw (PS) of the CD was threaded into the nail’s core until it made contact with the pin, thereby pushing the proximal fragment toward the distal one and enabling interfragmentary compression (Fig. 4F). The PS was tightened by the surgeon until full resistance to torque was achieved. Subsequently, the second locking screw was placed in the proximal fragment to maintain the achieved compression. The PS and the Steinmann pin were then removed, and a final locking screw was inserted in the proximal aspect of the nail, completing the fixation procedure. Finally, the CD and EIG were removed, and postoperative radiographic and CT images were obtained to assess femoral alignment and measure the residual fracture gap.

### Statistical analysis

Normality of the data was assessed using the Shapiro–Wilk test for each variable within each of the three groups. Statistical analysis of the variables was performed by calculating the mean and standard deviation after the assumption of normal distribution. Comparative analysis between pre‐ and postoperative time points for all variables was conducted using the paired *t*‐test. For comparisons between groups, the variation (Δ) of each variable was calculated as the difference between pre‐ and postoperative bone angle measurements. These Δ values were analysed using one‐way analysis of variance (ANOVA), followed by Tukey’s post hoc test for multiple comparisons. The effect size – representing the proportion of variance in the measured variable attributable to the treatment – was calculated using generalised eta squared (ges). Interpretation of the ges values followed the criteria established by Tomczak and Tomczak (2014): .1 ≤ ges ≤ .3 indicating a small effect, .31 ≤ ges ≤ .50 a medium effect and ges ≥ .51 a large effect. All statistical analyses were performed using R software, version 4.0.3 (R Core Team, R Foundation for Statistical Computing, Vienna, Austria), with a significance level set at 95% (*P* < .05).

## RESULTS

**FIG. 5:**
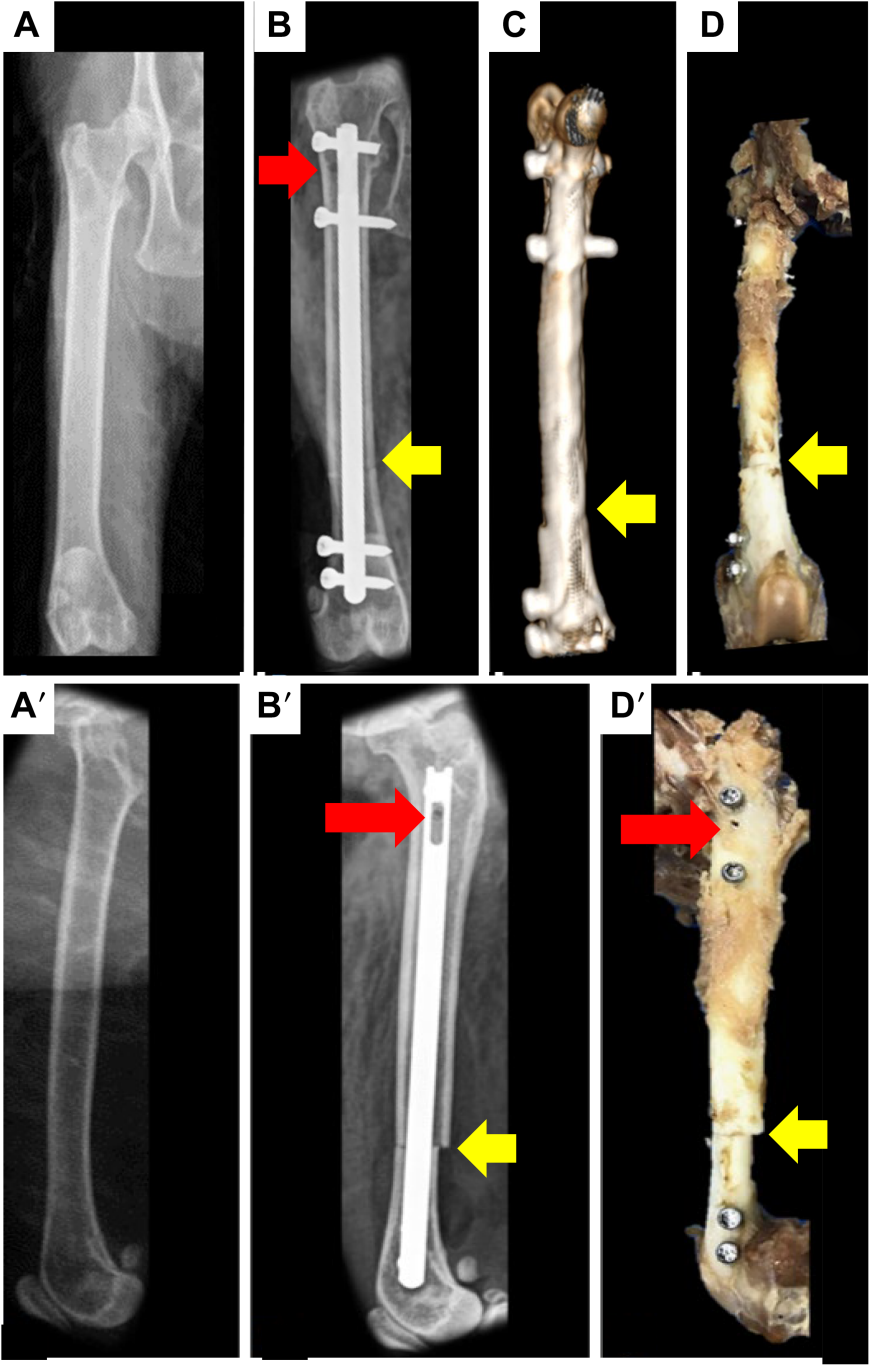
Pre‐ and postoperative visualisation and comparison of minimally invasive osteosynthesis using the dynamic compression angle‐stable interlocking nail (DCASIN) in ex vivo feline femurs. (A and A′) Pre‐osteotomy craniocaudal and mediolateral views. (B and B′) Postoperative craniocaudal and mediolateral views following DCASIN osteosynthesis, showing four angle‐stable locking screws, compressed bone fragments at the fracture site (yellow arrow), and the oblong hole of the DCASIN with the Steinmann pin perforation (red arrow) used to achieve dynamic compression. (C) Three‐dimensional computed tomography reconstruction after DCASIN osteosynthesis, highlighting the compressed osteotomy line (yellow arrow). (D and D′) Macerated femur following DCASIN fixation, demonstrating the lateromedial pin perforation (red arrow) and the resulting interfragmentary compression at the osteotomy site (yellow arrow).

Osteosynthesis using DCASIN was performed ex vivo on feline femurs (Fig. 5). The cadaver’s mean body weight was 4.0 kg (range: 3.5 to 5.0 kg). The femoral anatomical angles in the frontal plane, as well as the FTA in the transverse plane, following osteosynthesis with experimentally induced transverse fractures, were assessed. Regarding the aLDFA (Table 1), no significant differences were observed between pre‐ and postoperative measurements across the three groups, suggesting that MINO with DCASIN does not result in distal femoral deviation in the frontal plane. However, for the anatomical lateral proximal femoral angle (aLPFA) (Table 2), a significant difference was observed between pre‐ and postoperative values in the GMD, while the GPD and GDD femurs showed no significant changes. The effect size (ges) indicated a medium effect of the implant on this variation (Table 2). With respect to the FTA, all three groups demonstrated significant differences between pre‐ and postoperative measurements, with the GPD group showing the largest effect size (ges) associated with the implant (Table 3). The altered positioning of the greater trochanter within the same projection (Fig. 5A′,B′) subjectively illustrates the change in FTA. Finally, when comparing the three groups in terms of changes in the measured bone angles, no statistically significant differences were found, indicating that the fracture location was not associated with angular deviation (Table 4).

**Table 1:** Mean ± standard deviation (SD) of the anatomical lateral distal femoral angle (aLDFA) before and after osteosynthesis in ex vivo feline femurs with three fracture locations, treated using a dynamic compression angle‐stable interlocking nail via a minimally invasive approach

Group	Moment	Femurs	aLDFA ± SD (°)	Range (°)	P‐value	Ges^†^
GPD^‡^	Preoperative	10	90.47 ± 2.05	86.4 to 93,4	.576	.018
	Postoperative	10	89.95 ± 2.04	86.3 to 94.2		
GMD^§^	Preoperative	10	91.65 ± 2.17	88.3 to 95,8	.138	.118
	Postoperative	10	90.33 ± 1.59	88.2 to 93.8		
GDD^¶^ ^,^ ^†^	Preoperative	10	91.68 ± 1.57	89.8 to 95.2	.632	.013
	Postoperative	10	91.09 ± 1.57	85 to 96.3		

^†^ Generalised eta squared.

^‡^ Group proximal diaphysis.

^§^ Group mid‐diaphysis.

^¶^ Group distal diaphysis.

**Table 2:** Mean ± standard deviation (SD) of the anatomical lateral proximal femoral angle (aLPFA) before and after osteosynthesis in ex vivo feline femurs with three fracture locations, treated using a dynamic compression angle‐stable interlocking nail via a minimally invasive approach

Group	Moment	Femurs	aLPFA ± SD (°)	Range (°)	P‐value	Ges^†^
GPD^‡^	Preoperative	10	105.12 ± 4.9	95.9 to 112.3	.718	.007
	Postoperative	10	106 ± 5.79	95.8 to 114.9		
GMD^§^	Preoperative	10	105.32 ± 3.43	98.7 to 110.1	.001	.445
	Postoperative	10	110.95 ± 3.20*	105 to 115.8		
GDD^¶^	Preoperative	10	107.34 ± 3.74	101.8 to 114.5	.418	.002
	Postoperative	10	107.8 ± 7.55	96.9 to 119.9		

^†^ Generalised eta squared.

^‡^ Group proximal diaphysis.

^§^ Group mid‐diaphysis.

^¶^ Group distal diaphysis.

* Statistical difference (*P* < .05).

**Table 3:** Mean ± standard deviation (SD) of the femoral torsion angle (FTA) before and after osteosynthesis in ex vivo feline femurs with three fracture locations, treated using a dynamic compression angle‐stable interlocking nail via a minimally invasive approach

Group	Moment	Femurs	FTA ± SD (°)	Range (°)	P‐value	Ges^†^
GPD^‡^	Preoperative	10	25.12 ± 5.13	18.1 to 36	<.001	.655
	Postoperative	10	56.16 ± 15.99*	30 to 75.7		
GMD^§^	Preoperative	10	22.63 ± 4.88	12.4 to 29.9	.008	.385
	Postoperative	10	46.62 ± 22.06**	5.7 to 75.1		
GDD^¶^	Preoperative	10	34.09 ± 10.6	17.1 to 58.7	.035	.213
	Postoperative	10	57 ± 31.11***	10.8 to 91.6		

*, **, *** Statistical difference (*P* < .05).

^†^ Generalised eta squared.

^‡^ Group proximal diaphysis.

^§^ Group mid‐diaphysis.

^¶^ Group distal diaphysis.

**Table 4:** Mean ± standard deviation (SD) of the variations for anatomical lateral proximal femoral angle (aLPFA), anatomical lateral distal femoral angle (aLDFA) and femoral torsion angle (FTA) before and after osteosynthesis in ex vivo feline femurs with three fracture locations, treated using a dynamic compression angle‐stable interlocking nail via a minimally invasive approach

Angles	Group	Femurs	Mean ± SD variation (°)	Range (°)	P‐value	Ges^†^
aLPFA	GPD^‡^	10	2.3 ± 1.6	0.2 to 4.9	.75	.158
	GMD^§^	10	3.65 ± 2.36	0.1 to 7.3		
	GDD^¶^	10	4.42 ± 4.42	0.6 to 7.5		
aLDFA	GPD^‡^	10	1.54 ± 1.54	0.2 to 3.9	.30	.062
	GMD^§^	10	2.26 ± 2.26	0.3 to 5		
	GDD^¶^	10	1.51 ± 1.51	0.6 to 3.9		
FTA	GPD^‡^	10	31.04 ± 13.67	7.6 to 49.7	.74	.117
	GMD^§^	10	29.75 ± 13.21	13.5 to 53.1		
	GDD^¶^	10	40.37 ± 13.55	18.3 to 57		

^†^ Generalised eta squared.

^‡^ Group proximal diaphysis.

^§^ Group mid‐diaphysis.

^¶^ Group distal diaphysis.

Additionally, the interfragmentary compression achieved using the DCASIN was evaluated in feline femoral transverse fractures by measuring the residual fracture gap after osteosynthesis. All femurs (*n* = 30) demonstrated ideal or satisfactory compression outcomes, with residual gaps measuring less than 1 mm. No unsatisfactory gaps were observed, confirming the compressive capacity of the implant. There were no significant differences in mean gap measurements among the groups (Table 5).

**Table 5:** Mean ± standard deviation (SD) of residual fracture gap measurements after osteosynthesis in ex vivo feline femurs with three fracture locations, treated using a dynamic compression angle‐stable interlocking nail via a minimally invasive approach

Group	Femurs	Mean ± SD (mm)	Range (mm)	P‐value
GPD^†^	10	0.09 ± 0.1	0 to 0.3	.895 (vs. GMD^‡^)
GMD^‡^	10	0.07 ± 0.09	0 to 0.3	.972 (vs. GDD^§^)
GDD^§^	10	0.08 ± 0.08	0 to 0.3	.972 (vs. GPD^†^)

^†^ Group proximal diaphysis.

^‡^ Group mid‐diaphysis.

^§^ Group distal diaphysis.

## DISCUSSION

Dynamic compression in transverse fractures, although not essential to achieve fracture union (Tanveer et al., 2025), is a well‐established and desirable principle, as it favours primary bone healing and facilitates clinical recovery (Tseng et al., 2025; Ya’ish et al., 2011). Moreover, the MIO techniques may further improve outcomes by preserving the biological integrity of adjacent soft tissues and vascular supply (Déjardin et al., 2020; Maritato & Barnhart, 2020). Based on this rationale, DCASIN was developed to enable dynamic compression even when applied through a MINO approach (Dias et al., 2025). However, to date, no studies have evaluated either the implantation technique or the interfragmentary compression capacity of DCASIN in realistic long bone fracture models. Therefore, the present study aimed to address this gap by validating the DCASIN compression system. The findings demonstrated its ability to achieve effective cortical contact in all femurs after compression, even when applied via a MINO approach. Additionally, the study confirmed the feasibility of MINO with DCASIN, while emphasising the need for intraoperative attention to potential changes in FTA and aLPFA. These results support the initial hypothesis, confirming the capacity of DCASIN to generate dynamic compression under minimally invasive conditions and highlight the importance of anatomical landmarks observation to avoid postoperative angular deviations.

The interfragmentary compression is typically achieved through the use of dynamic compression plates (DCP) (Tseng et al., 2025; Ya’ish et al., 2011). However, other categories of implants can be adopted for this purpose. In human medicine, along with biological advantages, studies have demonstrated the mechanical advantages of compressive nails, which, in addition to the well‐established benefits of strategic position within the neutral axis and a high area moment of inertia, further favour the load sharing bone‐implant at the same time (Högel et al., 2013; Karakaşli et al., 2015). The recently developed DCASIN model may potentially facilitate the application of such principles in veterinary practice (Dias et al., 2025), supporting its application through a MINO approach. Based on the present results concerning compressive capacity, all feline femurs exhibited ideal or satisfactory postoperative gap measurements, considering that 1 mm could be a critical value to reduce the construct stiffness (Oh et al., 2010), and that larger gaps are associated with negative impacts on the bone healing process (Claes et al., 1997). In addition, human femurs treated with compressive nails exhibited an increased risk of non‐union when gaps were greater than 4 mm (Nakagawa et al., 2024). It is important to highlight that literature lacks the ideal measurement for residual gap for veterinary patients treated with dynamic compression nails and further clinical studies should be conducted to provide this information. Notably, the gap measurement considered the greatest linear distance between proximal and distal cortices on craniocaudal or mediolateral radiographs. In most cases, the craniolateral cortices showed close contact, while the caudomedial cortices were typically associated with wider gaps. This characteristic may potentially reflect expected anatomical variations in healthy animals related to physiological femoral procurvatum and varus (Beer et al., 2023) or indicate some inaccuracy of the implantation technique during surgery. Accordingly, nailing osteosynthesis is expected to modify and realign the anatomical axis of the bone (Déjardin et al., 2020), and intraoperative interfragmentary compression may contribute to the observed differences in cortical contact between bone surfaces. Nevertheless, a previous human study reported that a minimum of 50% cortical contact in compressive nailing osteosynthesis may be sufficient to promote bone healing (Högel et al., 2013), reinforcing the clinical viability of DCASIN based on the findings presented here.

In addition to its mechanical advantages, it is important to emphasise that DCASIN osteosynthesis was feasible using the MINO technique, offering biological benefits to the healing process (Déjardin et al., 2020; Guiot & Déjardin, 2011; Maritato & Barnhart, 2020). This feature may represent an advantage over the DCP or LC‐DCP, which generally compromise the biological environment due to the ORIF approach (Garofolo & Pozzi, 2013). However, the MIPO technique can be challenging, particularly with respect to iatrogenic bone deviations (Déjardin et al., 2020; Kim et al., 2017), as observed in the present protocol. Deviations in the frontal plane exhibited minimal variation between pre‐ and postoperative assessments. Specifically, the aLPFA changed only in the GMD, while aLDFA remained consistently unaltered across all three groups and both time points. The increase in aLPFA observed in the GMD was moderately associated with the DCASIN technique. Despite a mean increase of 5° in this variable, the authors hypothesise that such deviation is unlikely to be clinically significant, given the absence of evidence linking this degree of change to misalignment of the quadriceps extensor mechanism in either cats or dogs (Beer et al., 2023; Yasukawa et al., 2016), particularly for minor deviations such as those reported here. Nonetheless, significant variations were identified in the FTA.

FTA variations were observed across all three groups, particularly in the GPD group, where the effect of DCASIN was estimated to be large. In healthy cats, the normal FTA is approximately 25° (Santos et al., 2025), which aligns with the values recorded at the preoperative time point. The primary concern regarding femoral torsion lies in its direct association with patellar luxation in dogs (Longo et al., 2022). However, studies in felines have not identified a relationship between patellar luxation and femoral torsion, suggesting that mild FTA alterations are unlikely to result in clinically significant changes in limb alignment (Beer et al., 2023; Santos et al., 2025). Nevertheless, the observed moderate FTA variation highlights the risk of patellar luxation and underscores the need for heightened attention to transverse plane deviations during the MINO approach for DCASIN, as this may represent a limitation of the MIO technique, considering that similar alterations have been reported with other minimally invasive methods, such as plate fixation (Costa Junior et al., 2021; Peirone et al., 2020). This finding reinforces the importance of careful identification of anatomical landmarks during surgery (Kowaleski, 2020), particularly when adopting the interlocking nail technique, as this implant does not provide torsional control and surgeons play a crucial role in preventing torsional malalignment; thus they must remain vigilant to avoid such deviations (Déjardin et al., 2020; Kowaleski, 2020).

Limitations to our study include the ex vivo experimental model used for the implantation technique, which closely resembled the routine surgical approach; however, it may also be regarded as a limitation of the protocol. Additionally, a transverse osteotomy was employed to simulate a transverse fracture. Although this approach approximates the clinical scenario, it does not fully replicate real‐life cases, in which surrounding soft tissues and fibrosis can introduce challenges during surgery (Peirone et al., 2020) and may directly affect the ability to achieve interfragmentary compression. Furthermore, although radiographic assessment is the most appropriate method for measuring the residual gap in real clinical cases, it should be acknowledged as a limitation in the present ex vivo study, as magnification and projection errors could possibly have led to either overestimation or underestimation of the gap size. Therefore, future clinical trials involving naturally occurring fractures are encouraged to validate the results observed with DCASIN and to better characterise the potential limitations and bone deviations associated with its implantation in real patients.

The application of the MINO technique with DCASIN in feline cadaveric femurs demonstrated its ability to achieve satisfactory interfragmentary compression across all models evaluated, maintaining all fracture gaps below 1 mm. No changes in aLDFA were observed, regardless of fracture location, nor were there alterations in aLPFA in proximal and distal diaphyseal fractures. However, the technique was associated with changes in aLPFA in mid‐diaphyseal fractures, as well as alterations in femoral torsion across all groups assessed, highlighting the essential attention to anatomical landmarks during the surgical procedure. These findings encourage future clinical trials involving this nail model with dynamic compression and angle‐stable locking mechanisms.

## Author contributions

Matheus Nobile: Conceptualization; investigation; writing—original draft; project administration; data curation; validation; writing—review and editing; formal analysis (equal). Renato Barroco Neto: Conceptualization; investigation; project administration; data curation; validation; formal analysis (equal). Alefe L. C. Carrera: Investigation; writing—original draft; writing—review and editing; project administration; data curation; formal analysis (equal). Thiago A. S. S. Rocha: Writing—review and editing; methodology; validation; resources; supervision (equal). Vinicius S. V. Dias: Conceptualization; investigation; data curation; writing—review and editing; visualization (equal). Bruno W. Minto: Investigation; writing—review and editing; supervision; project administration; methodology (equal). Luis G. G. G. Dias: Funding acquisition; conceptualization; writing—review and editing; methodology; project administration; supervision; formal analysis (equal).

## Conflict of interest

Luis Gustavo Gosuen Gonçalves Dias and UNESP are co‐holders of a patent for the DCASIN and CD models (patent number BR 102018 016021 4), registered with the Brazilian National Institute of Industrial Property. The other authors declare no conflicts of interest.

## Funding

None.

## Data Availability

The data that support the findings of this study are openly available in the Zenodo repository at https://doi.org/10.5281/zenodo.15851200.
